# Biomarker Potential of Vimentin in Oral Cancers

**DOI:** 10.3390/life12020150

**Published:** 2022-01-20

**Authors:** Saie Mogre, Vidhi Makani, Swapnita Pradhan, Pallavi Devre, Shyam More, Milind Vaidya, Crismita Dmello

**Affiliations:** 1 Department of Veterinary and Biomedical Sciences, The Pennsylvania State University, University Park, PA 16802, USA; sum415@psu.edu; 2 Vaidya Laboratory, Advanced Centre for Treatment, Research and Education in Cancer (ACTREC), Tata Memorial Centre (TMC), Kharghar, Navi Mumbai 410210, India; vidhimakani@gmail.com (V.M.); swapnita.pradhan@gmail.com (S.P.); pallavi.devre17@gmail.com (P.D.); 3 F. Widjaja Foundation Inflammatory Bowel & Immunobiology Research Institute, Department of Medicine, Cedars-Sinai Medical Center, Los Angeles, CA 90048, USA; shyam.more@cshs.org; 4 Department of Neurological Surgery, Northwestern Medicine Lou and Jean Malnati Brain Tumor Institute, Robert H. Lurie Comprehensive Cancer Center, Feinberg School of Medicine, Northwestern University, Chicago, IL 60611, USA

**Keywords:** vimentin, biomarker, oral cancer

## Abstract

Oral carcinogenesis is a multistep process. As much as 5% to 85% of oral tumors can develop from potentially malignant disorders (PMD). Although the oral cavity is accessible for visual examination, the ability of current clinical or histological methods to predict the lesions that can progress to malignancy is limited. Thus, developing biological markers that will serve as an adjunct to histodiagnosis has become essential. Our previous studies comprehensively demonstrated that aberrant vimentin expression in oral premalignant lesions correlates to the degree of malignancy. Likewise, overwhelming research from various groups show a substantial contribution of vimentin in oral cancer progression. In this review, we have described studies on vimentin in oral cancers, to make a compelling case for vimentin as a prognostic biomarker.

## 1. Introduction

The cytoskeleton comprises microfilaments, intermediate filaments (IF), and microtubules. Together, the three filament systems operate as an integrated, dynamic network that is functionally regulated through their associated proteins to mediate cytoskeletal scaffolding [1]. Of the three types of cytoskeletal proteins, intermediate filaments are involved in an assortment of cellular functions, such as cell division and plasticity, motility, mechanical stress resistance, and organelle transport [2]. At least 65 genes encode the six major categories of intermediate filaments [3,4]. Type I and II intermediate filaments include acidic and basic keratins that are predominantly found in the epithelial cells. Vimentin and desmins are the Type III intermediate filaments that are primarily found in cells of mesenchymal origin and muscle cells, respectively; however, vimentin expression is also observed in other cell types, such as endothelial cells, macrophages, neutrophils, and lymphocytes as the only form of intermediate filaments [5]. Type III IF glial fibrillary acidic protein (GFAP) and peripherin are found solely in astrocytes and peripheral neurons. Type IV neurofilaments and α-internexin are present in neurons, whereas Type V IF neurofilaments include nuclear lamins. Nestins are the type VI IF and are detected in neuronal epithelial cells and in embryonic neurons [3]. IF-associated proteins (or IFAPs) organize intermediate filaments in bundles and networks. These include plectin, ankyrin, desmoplakin, and filaggrin [1].

Structurally, intermediate filament proteins share a central α-helical rod domain flanked by the non-α-helical N- and C-terminal end domains known as the head and the tail [6]. The central α-coiled rod domain of the individual molecules is further divided into the coil segments 1A, 1B, 2A, 2B1, and 2B2. The L1 linker segment links coil 1A and 1B, while the L12 links coil 1 and 2 [7]. A pre-coil domain (PCD) that does not engage in the coiled-coil formation precedes coil 1A. As a dimer, the vimentin rod is flanked by the flexible head domain on the left and the tail domain on the right [8]. Rod 1B assembles into A11 tetramer in an anti-parallel alignment of two parallel coiled-coil structures formed by the segments of rod 1. A vimentin monomer thus assembles itself into a homodimer that can later form tetramers and octamers [9]. 

Intermediate filaments, including vimentin, have functions distinct from those of the microfilaments and microtubules. Early research indicated that vimentin provides structural support to maintain cellular integrity and resistance to stress [10]. The structural and physiological functions of IFs are interconnected [6]. They are involved in wound healing by controlling fibroblast proliferation, TGFβ1-Slug signaling, collagen accumulation and EMT, proliferation [11], adhesion [12], migration and invasion [13,14], and as a positive regulator of stemness [15]. A survey of the Human Protein Atlas database showed vimentin expression in the majority of the tissues analyzed [16,17], wherein the multiple roles of vimentin in physiological and pathophysiological stress conditions are well established [18]. Homozygous deletion of vimentin (*Vim^−/−^*) in mice impaired the normal development of the mammary gland [15], glia [19], angiogenesis [20], and myelination of peripheral nerves [21]. In normal cells, vimentin synthesis occurs during embryogenesis in the primary streak stage, albeit restricted to the primary mesenchymal cells at this time, and it is associated with cell migration [22]. Vimentin is known to be involved in regulating actomyosin contractile force and can interact with the extracellular matrix to promote cell motility [23,24]. There is also evidence that interaction with vimentin may affect the function of chaperones [5]. One study reported that vimentin plays a role in the protection against misfolded proteins in the cells [17]. 

Although a majority of studies have described an intracellular role of vimentin, it is also noted to be present on the surface of the cells and in the extracellular matrix when secreted via the Golgi apparatus [25,26]. Additionally, vimentin controls cell proliferation, apoptosis, and differentiation. It promotes cell plasticity either by forming new cells through proliferation or by differentiating into new types of cells [17,27,28]. Furthermore, vimentin is also known to contribute to the aging process. Increased mRNA and protein expression are observed in senescent cells, suggesting a potential application of a vimentin variant as a marker for oxidative stress and aging [29]. Finally, vimentin-expressing cells possess a higher capacity to adapt to pathological conditions than those lacking vimentin. Thus, vimentin upregulation presents as an important drug target and a clinical biomarker [17]. Our previous work shows that vimentin is involved in reprogramming the epithelial state to a more mesenchymal state by controlling the expression of keratin pair of K5/K14 in an oral cancer-derived cell line [30,31]. Together, these studies suggest the diverse context-dependent structural and functional roles of vimentin.

## 2. Regulators of Vimentin

A complex transcriptional machinery regulates vimentin. In a comprehensive review, Satelli and Li [32] have described the various regulatory elements within its promoter region. These include TATA boxes, GC-boxes [33,34], and binding sites for transcription factors such as NF-ĸB, AP-1 containing the TGFβ1-response elements [33,35,36], PEA3 [37], Sp/XKLF [38], β-catenin/TCF4 [39,40,41], and ZBP-89 [38,42]. In addition to direct transcriptional regulation, epigenetic modification of vimentin expression has also been reported. Knockdown of SIRT1 (Sirtuin 1 histone deacetylase) decreased ZEB1 expression and subsequently of vimentin to suppress EMT in HNSCC [36]. Likewise, preventing DNA methylation by 5-aza-deoxycytidine in colon cancer cells dramatically increased vimentin mRNA expression [33]. These studies suggest various cell- and tissue-specific activators or repressors of vimentin expression.

Recent reports suggest that the post-transcriptional regulation of vimentin can also play a crucial role in cancer progression. For example, in a non-small cell lung carcinoma cell line, binding of NANOS3 protein to vimentin mRNA regulated the length of the poly(A) tail and prevented microRNA-mediated repression of vimentin, causing an increase in the invasive potential of these cells [37]. Furthermore, exosomes derived from hypoxic OSCC cells showed high levels of miR-21, which caused significantly enhanced Snail and vimentin expression in these cells [38]. However, further studies are required to adequately understand the role of microRNAs in the regulation of vimentin expression.

Vimentin is an excellent substrate for post-translational modifications (PTM) on account of its multiple domains and residues [6,7,8,9,32,43]. A thorough review by Snider and Omary has described several PTMs of vimentin, including but not limited to, phosphorylation, SUMOylation, and ADP-ribosylation, which were discovered by LC-MS-based analyses [44]. Interestingly, partial phosphorylation of vimentin by 14-3-3 can soften the filament to facilitate increased mobility of cancer cells [45]. Moreover, phosphorylation of vimentin on distinctive serine residues plays a role in a range of biological activities, such as cell motility, cytokinesis, IF assembly, and disassembly [46]. Nonetheless, while phosphorylation by several tyrosine kinases and SUMO 2/3 modifications of vimentin plays a key role in promoting cell growth and migration, mechanisms and pathophysiological consequences of SUMOylation, O-linked glycosylation, and other PTMs in cancers remain poorly understood [44,47,48,49].

## 3. Aberrant Expression of Vimentin in Premalignant Oral Lesions

Various studies have reported that the expression pattern of vimentin changes markedly between normal and cancerous epithelial tissues of the prostate, gastrointestinal tract, breast, central nervous system, and lung. While mechanisms of transcriptional and translational regulation of vimentin during cellular events leading to cancer progression are diverse [32,44], overexpression of vimentin is associated with a more metastatic and invasive phenotype in these cancers. In this review, we have focused on vimentin expression in premalignant and malignant oral lesions.

Vimentin expression was observed in the basal epithelial cells of benign oral buccal mucosa lesions showing lymphocyte infiltration, suggesting an association between inflammation and vimentin in the non-dysplastic lesions of the oral cavity [50]. Our group has also reported aberrant vimentin expression in premalignant oral lesions, such as leukoplakia and submucous fibrotic (SMF) tissues, as well as primary keratinocyte cultures isolated from these tissues. Interestingly, the percentage of vimentin-positive lesions was higher in clinically non-homogenous leukoplakia than in homogeneous leukoplakia [51]. Furthermore, increased vimentin protein and mRNA levels statistically correlated with the degree of disease progression from dysplasia to invasive carcinomas [51,52]. 

## 4. Role of Vimentin in Oral Cancer Progression

Immunohistochemical analysis of 227 oral tumors suggested a significant correlation of vimentin expression with various prognostic factors of OSCC, such as the tumor size, clinical stage, regional lymph node metastasis, local recurrence, and poor survival [51]. In addition, analysis of leukoplakia and OSCC epithelial tissue samples also revealed high vimentin and low E-cadherin expression [53,54].

Based on published studies, we may hypothesize that the increased vimentin levels can lead to a higher grade of oral malignancy; however, the mechanisms by which vimentin plays a role in tumor progression remain unclear. Our lab has previously shown that exogenous expression of vimentin alone is insufficient but requires an additional carcinogenic trigger to transform premalignant lesion-derived cells. However, overexpression of vimentin alone caused the acquisition of EMT and stemness-related changes [54]. Furthermore, overexpression of vimentin led to decreased expression of E-cadherin, while knockdown resulted in an increased level of a differentiation-specific marker involucrin, suggesting a role of vimentin in maintaining the dedifferentiated state of cells during cancer progression [30,54]. Additional studies with the vimentin knockdown OSCC-derived cells suggested a role of vimentin in modulating the expression of K5/K14, mediated partly through ΔNp63 to favor a dedifferentiated phenotype that can promote tumor progression [30]. Another study reported an inverse expression pattern of vimentin and β4 integrin to modulate cell motility by destabilizing β4 integrin-mediated adhesions in OSCC [55].

## 5. Vimentin in Late Stages of Oral Cancer

As a driver towards acquiring stemness-related signatures in premalignant oral cancer lesions, our lab has shown a critical role of vimentin in the development and progression of oral cancers [30,54]. Vimentin has been associated with poor prognosis in patients with higher histological degrees of OSCC malignancies [56]. While studies have documented statistical associations between higher vimentin expression and cancer progression through tumor stages and increased metastasis [51,56,57,58], its expression was not limited to the invasive front of tumor cells or other histological measures of invasiveness [59]. Nonetheless, Lazarevic and colleagues reported higher expression of EMT markers, including vimentin, in primary cell cultures derived from surgically resected margins compared to those from the tumor tissues obtained from six patients with OSCC [60]. The states wherein the lesions expressed altered E-cadherin and vimentin were referred to as undergone partial EMT by Wangmo and colleagues. These were clinicopathologically associated with poor survival for patients presented with primary OSCC, as determined by univariate Cox regression [61]. Similarly, Liu et al. have shown that among five EMT markers, Snail, Twist, E-cadherin, N-cadherin, and vimentin, vimentin is the most promising prognostic marker. This study was done in tongue squamous cell carcinoma patients using tissue microarray immunohistochemistry [62].

In addition, cell membrane β-catenin expression was significantly associated with vimentin in HPV-associated oropharyngeal squamous cell carcinoma [63]. In an OSCC-derived cell line Tca8113, treatment of exogenous TGFβ1 upregulated vimentin expression at both mRNA and protein levels. TGFβ1-dependent upregulation of vimentin was associated with increased migration and invasion of the OSCC cells; inhibiting TGFβ1 abrogated the migratory potential of these cells, suggesting a role of vimentin in driving TGFβ1-induced EMT in these cells [64]. Together, these studies suggest a role of elevated vimentin expression in the late stages of OSCC. 

## 6. Vimentin in Lymph Node Metastasis

An increasing number of studies have described the role of vimentin in lymphatic invasion and lymph node metastasis of oral, esophageal, gastric, prostate, and colorectal squamous cell carcinomas. Our lab has shown the role of aberrant vimentin expression in tumor cut margins to be significantly correlated to lymph node metastasis in OSCC [51]. Moreover, a transcriptomic study comparing HN12, an OSCC cell line derived from lymph node metastasis, to HN4, its non-metastatic equivalent, showed an 87-fold increase in vimentin expression. Incidentally, induction of EMT by TGFβ1 in the non-metastatic cell line upregulated the expression of vimentin [65]. Immunohistochemical analysis of OSCC showed significantly higher vimentin expression in lymph node metastasis. High vimentin mRNA expression was also significantly correlated with lymph node metastasis in squamous cell carcinomas derived from the sebaceous gland [65,66].

Jin and colleagues have also identified vimentin as an independent prognosticator of lymph node metastasis in esophageal squamous cell carcinomas (ESCC). Vimentin-positive ESCC cells exhibited increased incidences of lymph node metastasis, lymphatic invasion, and distal node metastasis [67,68]. While 74.2% of primary ESCC tumors showed elevated vimentin expression, the incidence of lymph node metastasis was reported in 71.9% of vimentin-positive tumors compared to 35.5% in vimentin-negative ESCC tumors. This increase in vimentin expression was also associated with a lower 5-year survival rate at 42.9% versus 66.1% of the patients (*p*-value = 0.0167) [68]. Furthermore, out of OSCC patients with malignant lesions with high vimentin expression, 60.7% presented with metastatic lymph nodes [57]. On the contrary, in a survey with a total of 60 OSCC patients with and without lymph node metastasis, Balasundaram and colleagues reported no significant difference in the degree of cytoplasmic vimentin expression in OSCC patients in the groups studied [69]. 

A study has also implicated the potential to target the cell surface domains of vimentin expressed concomitantly along with the stem cell markers CD44 and CD133 in the tumor-initiating metastatic pancreatic cancer cells derived from lymph nodes [70]. Together, while the mechanisms for high vimentin expression leading to increased lymph node metastasis remain unclear, increased expression can predict the behavior of OSCC tumors.

## 7. Vimentin in Angiogenesis

Post-translational modifications and functions of the cell-surface and extracellular vimentin have been studied extensively in processes involved in angiogenesis in both normal and cancer cells. After the initial discovery of the presence of vimentin on the surface of circulating tumor cells as reviewed by Pantel and colleagues [71], a group has reported a role of cell-surface vimentin in promoting endothelial tube formation by mediating a stable focal adhesion between the cell surface and extracellular matrix [72]. Assembly of the growth factor-induced and vimentin-associated matrix adhesions to plectin-αvβ3 integrin at the leading edge of actively migrating cells is required for the branching morphogenesis of primary endothelial cells [73]. In OSCC, the role of vimentin, along with vascular endothelial cadherin (VE-cadherin) and CD44 in vasculogenic mimicry (VM) formation in high-grade tumors, was noted. The study reported the presence of vimentin in detached and circulating tumor cells, suggesting acquisition of a cancer stem cell-like phenotype by these cells that co-expressed vimentin, CD44, and VE-cadherin at the periphery of tumor islands and invasive fronts. The observation further supported the finding that VM is present in 85.7% of high-grade OSCC tumors and suggests their role in the increased propensity towards metastasis [74]. In addition, OSCC cells overexpressing angiopoietin 2, a key regulator of angiogenesis, were also shown to have elevated markers of EMT, including vimentin. Upregulation of vimentin in angiopoietin 2-high OSCC cells coupled with a decreased expression of E-cadherin was associated with increased migration, invasion, and angiogenesis in nude mice [75]. 

## 8. Vimentin in Recurrence

While elevated vimentin expression is associated with poor prognosis in multiple cancers, studies have reported its association with recurrence, distant metastasis, and, consequently, a significantly lower disease-free survival [76,77]. With the exception of a report from our lab, wherein we showed that vimentin expression at the invasive front of OSCC tumor sections significantly correlated with local recurrence, vimentin has not been directly linked to untreatable, recurrent OSCC. However, as reviewed by Ling et al., several recent reports predicted that over a third of OSCC cases with type 3 EMT wherein elevated vimentin expression was noted are recurrent [51,78]. In agreement with these reports, a study described that 53% of the OSCC tumors overexpressing vimentin presented with disease recurrence and death [79]. Similarly, in a retrospective multivariate analysis of 274 patients with a history of resection of the primary oral cavity squamous cell carcinoma, a significant locoregional recurrence (hazard ratio of 6.59) was noted as one of the risk factors of developing distant metastasis [80]. Another study of 119 OSCC patients suggests a role of forkhead box protein M1 (FOXM1) to be associated with tumor recurrence. The knockdown of FOXM1 in OSCC cells also lowered vimentin expression and decreased proliferation and migration, potentially suggesting its role [81].

Consistent with findings from these studies in oral cancers, vimentin was shown as a prognosticator of disease recurrence with a risk ratio of 3.5 in advanced colorectal cancers [82]. A meta-analysis of 4118 non-small cell lung cancer cases revealed a significant odds ratio of 1.631 (*p*-value = 0.029) for disease recurrence to increased vimentin expression [83]. In addition, a significant decrease in vimentin expression and associated reduction in invasive capacity and disease recurrence was linked to inhibition of NF-κB-dependent TGFβ1 signaling in prostate cancer cells [84]. Contrary to these findings, another study showed no significant correlation with increased vimentin expression in colorectal patients with disease recurrence [85]. Nonetheless, these studies provide evidence for vimentin to be regarded as a predictor of advanced disease.

## 9. Vimentin in Therapy Resistance

Cisplatin and cetuximab resistance are significant challenges in chemoradiotherapy-resistant OSCC with poor prognoses [86,87]. Drivers of EMT, acquisition of cancer stem cell-like properties by tumor cells, miRNA deregulation, and cancer cell-derived extracellular vesicles (EV) have all been reported to play a pivotal role in the acquisition of drug resistance. In a spheroid model of OSCC, development of gradual EMT, acquisition of cancer stem cell markers, and activation of p38 MAPK-Hsp27 axis were observed during the development of drug resistance [88]. Increased EVs containing higher cisplatin levels than intracellular concentrations were also observed in cisplatin-resistant OSCC-derived cell lines [87]. 

Kirave and colleagues have demonstrated a role of a miRNA, miR-155, to transfer cisplatin resistance to cisplatin-responsive OSCC cells via exosomes to initiate EMT-dependent acquisition of resistance [89]. Similarly, another miRNA, miR-619-5p, was shown to be significantly downregulated in cisplatin-resistant OSCC. The authors showed that overexpression of miR-619-5p significantly blocked the induction of EMT, as assessed by the expression of vimentin and E-cadherin in cisplatin-resistant cells generated by gradually exposing HN6 and CAL27 OSCC cells with increasing doses of cisplatin [90]. Furthermore, loss of EGFR with elevated expression of EMT markers, including vimentin, Snail, and N-cadherin, have been noted in high-grade invasive OSCC cells that have developed resistance to cetuximab therapy [91]. In a study of OSCC tumor samples, the miR-200 family was significantly downregulated. Downregulation of miR-200 is associated with increased vimentin expression and decreased E-cadherin expression towards the induction of EMT [92,93]. Ghosh and colleagues have identified a set of six miRNAs, miR-130b, miR-134, miR-149, miR-491, miR-181d, and miR-146b, that may impart cancer stem cell-like properties and induce EMT in cisplatin-resistant OSCC-derived cell lines [94]. In addition, a study has defined a role of OSCC-derived cancer stem cells (CSCs) in resistance to therapy and subsequent recurrence. The authors showed the presence of two distinct CSC populations within the tumor: one that retained epithelial characteristics and one that resembled a mesenchymal, migratory phenotype (CD44^high^ESA^low^) and showed higher vimentin expression [95]. Interestingly, a study with 20 treatment-naïve head and neck cancer patients treated with cetuximab showed elevated markers of EMT early in the clinical course of drug treatment [96], highlighting the need for additional studies in the area to delineate the role of vimentin towards chemoresistance.

## 10. Association of Oral Cancer Risk Factors with Vimentin

Tobacco smoking, alcohol consumption, and HPV infection are well-known risk factors of oral cancer [97]. Studies have shown the association of these risk factors with vimentin expression. In 202 oropharyngeal squamous cell carcinoma (OPSCC) patients, vimentin expression was found in tumor-associated stromal cells. The strongest expression of vimentin and β-catenin was associated with HPV-positive OSCC tumors compared to the HPV-negative tumors. The same patient cohort showed a significant association of β-catenin and vimentin expression in patients with no history of heavy alcohol use. This association was not seen in patients who consumed alcohol. Similar findings were also reported by Bagnardi et al., where heavy alcohol use was associated with the development of HPV-negative OPSCC [98]. On the other hand, among oral squamous cell carcinoma patients (*n* = 85), no significant association was seen between vimentin expression and smoking or alcohol use [65]. This suggests that the association between vimentin and risk factors may show variation depending upon the site of origin of cancer. Furthermore, it will be interesting to investigate if any of these risk factors directly regulate levels of vimentin in oral cancer. 

## 11. Concluding Remarks

**Figure 1:**
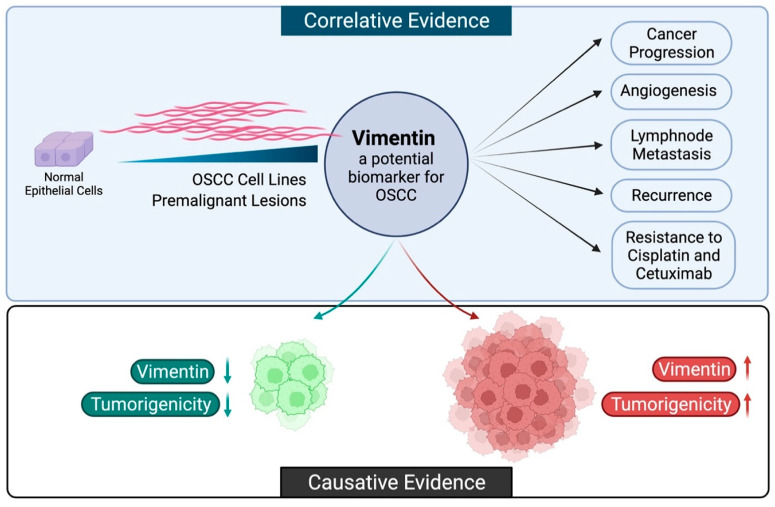
The biomarker potential of vimentin. The correlative and causative evidence described in this review article suggests the potential of vimentin as a biomarker in early and late events of OSCC. Vimentin is aberrantly expressed as oral epithelial cells transform and acquire malignant potential. High vimentin expression is correlated to increased progression, angiogenesis, metastasis, disease recurrence, and resistance to chemotherapy (Created with BioRender. Available online: https://biorender.com/, accessed on 10 December 2021).

The literature cited here shows a high promise in investigating vimentin as a potential biomarker candidate for oral cancer, since its expression correlates as well as contributes to the process of oral oncogenesis (Figure 1). We and others have shown that vimentin positivity in the late stages of oral cancers correlates with poor prognosis. Our studies on a premalignant oral cancer cell line showed that forced expression of vimentin in the early stages of cancer is advantageous for the transformation of the cancer cell. Collectively, vimentin has emerged as one of the drivers of the critical events and is widely regarded to be beneficial for tumor progression. Nonetheless, more studies correlating vimentin expression in early and late cancerous lesions of the oral cavity to the disease free-survival in a large number of patients are required to establish vimentin as a marker for poor prognosis in oral cancers. In parallel, extensive studies are needed to characterize the role of vimentin in resistance to chemotherapy.
